# A real-time PCR assay to estimate *Leishmania chagasi* load in its natural sand fly vector *Lutzomyia longipalpis*

**DOI:** 10.1016/j.trstmh.2008.04.003

**Published:** 2008-09

**Authors:** Shalindra Ranasinghe, Matthew E. Rogers, James G.C. Hamilton, Paul A. Bates, Rhayza D.C. Maingon

**Affiliations:** a Centre for Applied Entomology and Parasitology, Institute of Science and Technology in Medicine, Keele University, Staffordshire ST5 5BG, UK; b Department of Parasitology, Faculty of Medical Sciences, University Sri Jayewardenepura, Gangodawila, Nugegoda, Sri Lanka; c Liverpool School of Tropical Medicine, Pembroke Place, Liverpool L3 5QA, UK; d Department of Immunology, Imperial College of Science, Technology and Medicine, London, Norfolk Place, London W2 1PG, UK

**Keywords:** *Leishmania*, Sand fly, PCR, Sensitivity and specificity, Transmission, Epidemiology

## Abstract

*Leishmania chagasi*, transmitted mainly by *Lutzomyia longipalpis* sand flies, causes visceral leishmaniasis and atypical cutaneous leishmaniasis in Latin America. Successful vector control depends upon determining vectorial capacity and understanding *Leishmania* transmission by sand flies. As microscopic detection of *Leishmania* in dissected sand fly guts is laborious and time-consuming, highly specific, sensitive, rapid and robust *Leishmania* PCR assays have attracted epidemiologists’ attention. Real-time PCR is faster than qualitative PCR and yields quantitative data amenable to statistical analyses. A highly reproducible *Leishmania DNA polymerase* gene-based TaqMan real-time PCR assay was adapted to quantify *Leishmania* in sand flies, showing intra-assay and inter-assay coefficient variations lower than 1 and 1.7%, respectively, and sensitivity to 10 pg *Leishmania* DNA (∼120 parasites) in as much as 100 ng sand fly DNA. Data obtained for experimentally infected sand flies yielded parasite loads within the range of counts obtained by microscopy for the same sand fly cohort or that were around five times higher than microscopy counts, depending on the method used for data analysis. These results highlight the potential of quantitative PCR for *Leishmania* transmission studies, and the need to understand factors affecting its sensitivity and specificity.

## 1 Introduction

The leishmaniases are a set of diseases caused by *Leishmania* parasites, which affect more than 2 million people in over 88 tropical and Mediterranean countries. Resistance to first- and second-line chemotherapy, particularly in regions of intense *Leishmania* transmission, has been reported (Mittal et al., 2007). *Leishmania* parasites are transmitted to sylvatic or peridomestic mammalian reservoir hosts and to humans by blood-feeding female sand flies.

*Leishmania* real-time PCR assays for estimating relative loads within vertebrate hosts have been developed, based upon *Leishmania* small ribosomal subunit, DNA polymerase or glucose-6 phosphatase genes (Bossolasco et al., 2003, Bretagne et al., 2001, Mary et al., 2004, Nicolas et al., 2002, Wortmann et al., 2001). These PCR studies have indicated that *Leishmania* load influences clinical outcome and that low levels of parasitaemia or clearance are associated with either cure or fewer relapses in HIV–*Leishmania* co-infection. In canine leishmaniasis, the quantity of *Leishmania* DNA correlates with parasite density in the bone marrow, blood, skin or urine, and often with severity of clinical symptoms (Manna et al., 2006, Solano-Gallego et al., 2007). Furthermore, Svodová et al. (2003) demonstrated *Le*. *tropica* transmission to its *Phlebotomus sergenti* sand fly vector from asymptomatic ‘reservoir’ black rats using quantitative PCR. Thus, real-time PCR offers a feasible approach to follow *Leishmania* infection time course, parasite clearance and tissue tropism.

Natural infection rates in sand flies are traditionally estimated by microscopic identification of *Leishmania* within dissected sand fly guts and/or parasite isolation from dissected sand flies in vitro or in vivo. However, these methods are time- and labour-consuming, especially when considering the low infection prevalence found in most endemic foci (Ashford et al., 1991). A number of *Leishmania*-DNA-based PCR assays, with differing sensitivities and specificities, have been applied to studies of sand fly natural infection rates (Aransay et al., 2000, Córdoba-Lanús et al., 2006). *Leishmania* species typing through RFLPs, hybridisation or sequencing of amplified *Leishmania* DNA, with species-specific PCR primers, has also been reported (Azizi et al., 2006, Garcia et al., 2007, Jorquera et al., 2005, Martin-Sánchez et al., 2006).

Two drawbacks of end-point, compared with real-time, PCR are that the former is only qualitative and that it is still time-consuming, as PCR cycling time adds to the time required for visualisation of PCR products run in agarose gels. In real-time PCR assays, the PCR products are ‘visualised’ in real time and are also quantifiable, allowing statistical testing of the reproducibility and significance of results obtained. Furthermore, although initially more costly (i.e. for equipment and reagents), than end-point PCR, real-time PCR is significantly less time-demanding, reducing the overall research cost in the long term.

It is important to quantify *Leishmania* in sand flies to evaluate relative *Leishmania* development efficiencies between different sand fly species that transmit the same parasite species. These differences may account for vectorial capacity differences, which in turn could contribute to observed epidemiological differences between visceral leishmaniasis foci (Montoya-Lerma et al., 2003). It is also recognised that one major difference between natural and experimental infections is that the true parasite infective dose probably consists of 1–1000 metacyclics in natural conditions but several million in experimental infections (Rogers et al., 2004, Warburg and Schlein, 1986). Consequently, real-time PCR would allow more accurate determination of natural infection doses. Numerous reports have established that effective parasite dose egested at the vertebrate host biting site determines antigen concentration and distribution, and these in turn influence the timing and type of immune responses and hence clinical outcome (Lira et al., 2000). However, to date there are no published studies in which *Leishmania* load has been estimated within sand flies using as accurate a method as real-time PCR (Gómez-Saladín et al., 2005). This article reports the application of a TaqMan real-time PCR assay to quantify *Leishmania* within sand flies based on the *Le*. *infantum* single copy *DNA polymerase α* and the *Lutzomyia longipalpis periodicity* genes.

## 2 Materials and methods

### 2.1 *Leishmania* and sand fly maintenance

*Lutzomyia longipalpis* sand flies (Diptera: Psychodidae) from Jacobina, Bahia State, Brazil, were reared at 22–27 °C, 60–70% relative humidity and 12:12 (L:D) photoperiod, as described by Modi and Tesh (1983). Newly emerged flies were fed on 70% (w/v) sucrose solution ad libitum before processing for DNA extraction. *Leishmania infantum* (MHOM/BE/67/ITMAP263), a reference strain used in other PCR assay development studies (Noyes et al., 1996), was selected to develop the real-time PCR assay within sand flies. Promastigotes were cultured in HOMEM at 26 °C as described (Berens et al., 1976).

### 2.2 Sand fly experimental infection

Female *Lu. longipalpis* sand flies (∼125 flies in a cage, 5 d after mating) were fed on fresh rabbit blood seeded with *Le*. *infantum* amastigotes (2 × 10^6^/ml) through a chick skin membrane feeding apparatus (Rogers et al., 2002). Amastigotes were obtained from *Le*. *infantum*-infected BALB/c mouse spleen homogenates in M199 medium supplemented with 10% (v/v) heat-inactivated fetal calf serum, B and E vitamins (Gibco, Invitrogen Corp., Paisley, UK) and 25 μg/ml gentamycin sulphate (Sigma-Aldrich Co., Cambridge, UK) at pH 7.2. To prevent premature mortality, flies were allowed to defecate onto filter paper inside the cage but were prevented from laying eggs by continuous saturated sucrose feeding and by withdrawing oviposition substrates. Twelve infected flies [13 d post-infection to allow metacyclogenesis (Rogers and Bates, 2007)] were dissected and parasite numbers were estimated by microscopic examination of gut homogenates using a haemocytometer, as detailed by Rogers et al. (2002). Six infected flies from the same infection cohort were individually stored in 2 ml 96% (v/v) ethanol at room temperature, and were used for *Leishmania* quantitation by real-time PCR.

### 2.3 DNA isolation

Cultured *Leishmania* promastigotes (10^8^) were washed in buffered saline before DNA isolation, as described by Campos-Ponce et al. (2005). Individual female sand flies were placed onto 3MM Whatmann filter paper to allow the ethanol to evaporate for a few minutes before DNA extraction. An SDS-potassium acetate method (Collins et al., 1987) was used (average yield per fly obtained: 2040 ng DNA). DNA concentrations and enrichment relative to protein were determined at 260/280 nm in a Biophotometer (Eppendorf UK, Cambridge, UK). Sand fly samples were tested for amplification with microsatellite LIST MS6-001 PCR primers (GenBank accession no. **AF411613**; see Table 1), before storage at 4 °C.

**Table 1:** Qualitative and quantitative PCR primers and conditions

	Target	Forward primer^a^	Reverse primer^a^	Probe^a^	Conditions	Reference
*Leishmania*	KDNA 120 bp	CCTATTTTACACCAACCCCCAGT	GGGTAGGGGCGTTCTGCGAAA	End-point PCR	1.25 mmol/l MgCl_2_; 1 min, 94 °C; 40 cycles – 30 s, 94 °C + 30 s, 58 °C + 30 s, 72 °C	Nicolas et al., 2002

	DNA polymerase α 90 bp	TGTCGCTTGCAGACCAGATG	GCATCGCAGGTGTGAGCAC	5′-FAMCAGCAACAACTTCGAGCCTGGCACC-3′-TAMRA	3 mmol/l MgCl_2_; 5 min, 95 °C; 50 cycles – 15 s, 95 °C + 1 min, 65 °C	Bretagne et al., 2001

*Lu*. *longipalpis*	MSLIST6001 150 bp	AAAGGGTGCGAAGTTATTGC	GGGTGGGTTGGACATTCTAC	End-point PCR	2.5 mmol/l MgCl_2_; 1 min, 95 °C; 6 cycles – 30 s, 95 °C + 30 s, 53 °C + 45 s, 72 °C; 26 cycles – 30 s, 92 °C + 30 s, 53 °C + 55 s, 72 °C; 30 min, 72 °C	Watts et al., 2001

	*periodicity* 80 bp	ATTTCTTTTCCTTAGGACCATCGA	TAGGACATCTTCGGAAAATTGTTG	5′-AMTCCTCASAGTCTTTGCATCCACGTTGGTT-3′-TAMRA	3 mmol/l MgCl_2_; 5 min, 95 °C; 50 cycles – 15 s, 95 °C + 1 min, 65 °C	Bauzer et al., 2002

a DNA sequences are given in the standard 5′–3′ direction.

### 2.4 Qualitative PCR

End-point PCR was visualised on ethidium-bromide-stained 1.75% (w/v) wide range agarose (50–1000 bp; Sigma-Aldrich) gels to test whether isolated DNA was amplifiable and to determine the robustness, specificity, relative amplification efficiencies and lack of cross-inhibition of real-time PCR primers. Serial dilutions of *Leishmania* and/or sand fly DNA (100 ng–1 pg), were amplified with 100 pm PCR primers in 75 mmol/l Tris-HCl (pH 8 at 25 °C), 20 mmol/l (NH_4_)_2_SO_4_, 0.01% (v/v) Tween-20, 200 μM each deoxynucleotide triphosphate (dNTP), 1.25 units Thermoprime plus DNA Polymerase (Reddymix, ABgene, Epsom, UK) and optimum MgCl_2_ concentration in a final volume of 10 μl.

PCR primers suitable for TaqMan probe real-time PCR based on the *Lu*. *longipalpis periodicity* (*per*) gene sequence (GenBank accession no. **AF446142**; Bauzer et al., 2002) were designed using the Primer Express 2.0 software (Perkin-Elmer, Applied Biosystems, Foster City, CA, USA). Other sand fly and *Leishmania* PCR primers used with PCR/cycle conditions, amplicon sizes and sources are listed in Table 1.

### 2.5 Quantitative real-time PCR

*Leishmania DNA polymerase* primers described by Bretagne et al. (2001) and newly designed sand fly *per* primers were independently optimised for relative primer and magnesium concentration using 100 ng *Le*. *infantum* DNA and 250 nmol/l *Leishmania* TaqMan probe or sand fly *per* TaqMan probe, respectively, in an Applied Biosystems ABIPRISM 7000 amplification and fluorescence detection system. Optimised 20 μl PCR reactions contained 900 nmol/l each of the appropriate forward/reverse primer pair, 250 nmol/l *Leishmania* or sand fly TaqMan probe, 10 μl Universal TaqMan master mix (catalog 4304437, Applied Biosystems), and either 10-fold serial dilutions (100 ng–1 pg) of *Leishmania* DNA or sand fly DNA, 10-fold dilutions *Leishmania* DNA spiked over a fixed amount of sand fly DNA (100 ng) or DNA (average 130 ng per fly) from an experimentally infected sand fly. Reactions were pre-incubated at 50 °C for 2 min for uracyl-N-glycosylase activation, followed by denaturation/DNA polymerase activation at 95 °C for 10 min, and 40 amplification cycles, each of 15 s at 95 °C plus 1 min at 60 °C. Four independent assays were run on different days, each consisting of *Leishmania* DNA polymerase and *Lu*. *longipalpis* triplicate samples separately amplified in a single PCR plate. Negative (no template) controls, cross-amplification controls (*Leishmania* primers on 100 ng sand fly DNA and sand fly primers on 100 ng *Leishmania* DNA), and positive controls (100 ng *Leishmania* or *Lu*. *longipalpis* DNA), were included in each reaction plate.

### 2.6 Data analysis and statistical methods

The real-time PCR detection threshold was set at 10 times the standard deviation above the mean baseline fluorescence calculated from 2–12 cycles in the exponential phase. For each sample, mean fractional cycle numbers corresponding to the first amplification above the threshold value (threshold cycle, Ct) were used to obtain separate standard negative linear regression curves. Mean Ct values were plotted against the logarithm (base 10) of the DNA quantity of *Le. infantum DNA polymerase* and *Lu*. *longipalpis per* genes.

Input *Leishmania DNA polymerase* gene copy numbers were obtained using the absolute quantification method by interpolation of sample mean Ct values in the *Leishmania DNA polymerase* amplification standard curve. Reproducibility of the results was assessed through estimations of mean values, SDs and intra-assay and inter-assay variation coefficients (from raw Ct values) for four (*Leishmania DNA polymerase*) or five (sand fly *per*) independent repeat runs. One of the samples consisting of equal amounts of sand fly and *Leishmania* DNA (corresponding to 100 ng of each DNA) was used as control in a comparative method that related *Leishmania DNA polymerase* PCR signals to *Lu. longipalpis per* gene PCR signals (as reference) taking into account the efficiencies of both PCR primer sets. The following equation for obtaining the ratio relative to the amount of *Leishmania* DNA in the control (R) was used:

$$R=\frac{{\left(E_{Leishmania\;DNA\;polymerase}\right)}^{\Delta Ct\;DNA\;polymerase\;(\text{control}-\text{sample})}}{{\left(E_{Sand\;fly\;periodicity}\right)}^{\Delta Ct\;DNA\;periodicity\;(\text{control}-\text{sample})}}$$

where E is the amplification efficiency obtained from the linear regression standard curve using E = 10^−1/slope^ (Pfaffl, 2001).

Conversion of DNA amounts to *Leishmania* parasites, based on the size of the sequenced *Leishmania major* haploid genome (33.6 MB, 72.5 fg for its diploid genome) plus an estimated ∼15% kDNA (∼10.9 fg) yielded ∼83.4 fg total DNA for a single parasite. Based on these considerations, 10 pg *Leishmania* DNA represents ∼120 *Leishmania* parasites in this report.

## 3 Results

### 3.1 Specificity, sensitivity and reproducibility of the assays

Despite copy number differences, when tested by qualitative PCR, *Leishmania DNA polymerase* and kinetoplast origin primers (listed in Table 1) displayed similar sensitivity and specificity in amplifying an expected single product only in the presence of *Leishmania* DNA (10 pg to 100 ng) (Supplementary Figure 1). *Lutzomyia longipalpis* DNA (100 ng) did not interfere with *Leishmania DNA polymerase* gene amplification (water or sand fly DNA alone were negative) (Supplementary Figure 2A). *Lutzomyia longipalpis per* primers also showed similar specificity and sensitivity, producing the expected 80 bp product when tested by end-point PCR (Supplementary Figure 2B; data not shown).

**Figure 1:**
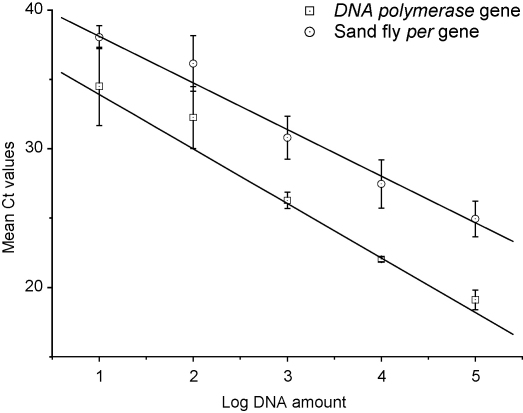
Standard curves for quantification of *Leishmania DNA polymerase* and *Lutzomyia longipalpis periodicity* gene input copies. () Mean *Leishmania DNA polymerase* Ct values ± 1 SD from independent experiments of three replicates of 10-fold serial dilutions of *Le. infantum* DNA in molecular biology grade (MBG) water, tested on different days, were plotted against the logarithm of the DNA amount (100 ng to 10 pg per reaction). Slope = –4.101; intercept = 39.139; *r*^2^ = 0.9821; efficiency = 10^−1/slope^ = 1.7533. () Mean *Lu*. *longipalpis per* Ct values ± 1 SD from five independent experiments of three replicates of 10-fold serial dilutions of sand fly DNA in MBG water, tested on different days, were plotted against the logarithm of the DNA amount (100 ng to 10 pg per reaction). Slope = –3.486; intercept = 41.936; *r*^2^ = 0.9799; efficiency = 10^−1/slope^ = 1.93581. Ct: the cycle number at which fluorescence rises significantly above the background fluorescence.

Figure 1 shows the sensitivity of the real-time PCR assays (10 pg, 10^5^ range), defined as the lowest DNA amount yielding amplification signals in all three replicates. This sensitivity was similar to that shown by qualitative PCR when tested using serial dilutions of *Leishmania* or *Lu*. *longipalpis* DNA of known concentrations determined by spectrophotometry (compare Figure 1 with Supplementary Figure 1A).

The reproducibility of both real-time PCR assays was tested running triplicate samples with standard curve dilutions, and controls on the same plate and on different days. The SDs obtained for the standard curves (*per* gene 0.83–2.01; *DNA polymerase* gene 0.18–2.82) are shown in Figure 1. Table 2 lists the inter-assay variation coefficients (0.01–0.11%) for *Leishmania* detection in the presence and absence of sand fly DNA. High amounts of sand fly and *Leishmania* DNA (100 ng each; see Table 2) appeared to inhibit amplification, whereas highly diluted *Leishmania*/sand fly DNA samples (10–100 pg; see Table 2) resulted, mostly, in *Leishmania* DNA overestimations, (especially for *Leishmania*/sand fly mixed samples). Determinations within the *Leishmania* 10–10 000 pg range in *Leishmania*-spiked 100 ng sand fly DNA samples were more consistent with optical-density-derived *Leishmania* DNA amounts than those for *Leishmania*/sand fly mixed DNA samples (see Table 2).

**Table 2:** Reproducibility of quantification of *Leishmania* in sand fly DNA samples

*Le. infantum* DNA/pg^a^	Mean Ct ± SD			Inter-assay variation coefficient	Estimated *Leishmania* DNA (pg)^e^	
	*Leishmania*^b^	*Leishmania*-spiked sand fly DNA^c^	*Leishmania*–sand fly mixed DNA^d^		*Leishmania*-spiked sand fly DNA	*Leishmania*–sand fly mixed DNA
10	34.50 ± 2.82	33.67 ± 0.20	29.83 ± 0.20	0.08	22	186
100	32.26 ± 2.23	32.45 ± 0.33	26.65 ± 0.12	0.11	43	1110
1000	26.28 ± 0.58	25.89 ± 0.10	24.48 ± 0.15	0.04	1700	3750
10 000	22.03 ± 0.18	21.67 ± 0.08	21.78 ± 0.62	0.01	18 200	17 100
100 000	19.11 ± 0.70	19.33 ± 0.11	20.28 ± 0.41	0.03	67 700	39 700

Ct: threshold cycle value, the cycle corresponding to the first noticeable fluorescence rise above the background fluorescence.

a DNA amount in 10-fold serial dilutions from a 100 ng/μl stock determined by spectrophotometry.

b Mean Ct values from four independent runs each of triplicate samples of 10-fold serially diluted DNA in molecular biology grade (MBG) water.

c Mean Ct values from one run of triplicate samples of 10-fold serially diluted DNA spiked onto 100 ng sand fly DNA samples.

d Mean Ct values from one run of triplicate samples of 10-fold serially diluted solution containing equal amounts of *Leishmania* and sand fly DNA.

e Mean Ct values (columns c and d) were related to a standard curve constructed with the mean Ct values for *Leishmania* DNA serially diluted in MBG water (Figure 1).

### 3.2 Estimation of *Leishmania* parasite loads in experimentally infected sand flies

Results obtained by real-time PCR using both the standard curve absolute quantification method and a relative quantification method to estimate the number of *Leishmania* parasites in experimentally infected sand flies are shown in Table 3. Low intra-assay variation coefficients were estimated for the sand fly *per* (0.0003–0.001%) and the *Leishmania DNA polymerase* (0.0004–0.005%) genes (see Table 3). Parasite number estimates based on the standard curve (see Figure 1) for the *Leishmania DNA polymerase* gene (six flies; mean 103 642; range 25 739–167 306) were three times higher than those obtained by microscopic examination of dissected gut homogenates of separate sand flies from four similarly infected cohorts (12 flies; mean 75 435; range 600–330 330) (Supplementary Table 1). *Leishmania* loads per fly calculated by the Pfaffl (2001) relative quantification method were five times higher than those obtained using the absolute standard curve method (six flies; mean 574 547; range 130 694–1 497 239) (see Table 3).

**Table 3:** Quantification of *Leishmania* in experimentally infected *Lutzomyia longipalpis* sand flies

Sand fly number	Sand fly *periodicity*^a^		*Leishmania DNA polymerase*^b^		*Leishmania* parasites/fly^c^	
	Mean Ct ± SD	Intra-assay variation coefficient	Mean Ct ± SD	Intra-assay variation coefficient	Absolute method^d^	Relative method^e^
1	26.59 ± 0.03	0.001	27.10 ± 0.06	0.002	160 778	1 497 239
2	25.67 ± 0.06	0.002	30.36 ± 0.02	0.0004	25 739	130 694
3	25.38 ± 0.06	0.002	28.11 ± 0.03	0.0008	91 207	381 810
4	25.61 ± 0.01	0.0003	28.78 ± 0.17	0.005	62 670	305 078
5	25.32 ± 0.06	0.002	27.71 ± 0.10	0.003	114 149	459 405
6	25.32 ± 0.03	0.002	27.03 ± 0.02	0.0007	167 306	673 054

Ct: threshold cycle value, the cycle corresponding to the first noticeable fluorescence rise above the background fluorescence.

a *Lu*. *longipalpis per* gene real-time PCR.

b *Le. infantum* DNA polymerase α gene real-time PCR.

c It was estimated that one parasite contained ∼83.4 fg total DNA.

d Mean Ct values for *Leishmania DNA polymerase* amplification in infected flies (b), were related to the mean Ct values (four independent experiments) obtained for 10-fold serially diluted *Leishmania* DNA in molecular biology grade (MBG) water (Figure 1).

e The method developed by Pfaffl (2001) was used. Mean Ct values for *Leishmania DNA polymerase* gene amplification in infected flies (b) were related to mean Ct values for sand fly *per* gene (a), and to the *Leishmania DNA polymerase* and sand fly *per* PCR signals obtained for a sample containing 100 ng sand fly and 100 ng *Leishmania* DNA used as control.

## 4 Discussion

*Leishmania* quantification by PCR yields copies of the target template input that the researcher can relate to parasite counts based on: (1) the number of target gene copies present in the *Leishmania* genome; (2) the accuracy of determination of *Leishmania* DNA or parasite numbers by an independent method; (3) sample factors, including total and target DNA concentration and presence of inhibitors; and (4) the assumed DNA amount per *Leishmania* parasite. The *Leishmania* assay used in the present study simplifies the stoichiometry between *Leishmania* parasite numbers and copies of target gene input to 1:1 as it is based on the single copy *DNA polymerase α* gene. In addition, amplification measurements based on hybridisation of an internal TaqMan probe provided assay specificity. Prior to quantitative PCR, PCR primers including the newly designed reference *Lu*. *longipalpis periodicity* primers for relative quantification were optimised by qualitative end-point PCR for efficiency, specificity and sensitivity.

The present assay showed intra- and inter-assay variation coefficients either lower than or comparable to *Leishmania* PCR assays reported (Bretagne et al., 2001, Manna et al., 2006, Nicolas et al., 2002, Wortmann et al., 2001). Indeed, high reproducibility was maintained despite the relatively poor accuracy of DNA amount estimates for low DNA concentrations determined by optical density and PCR depression observed at high sample DNA concentrations. Owing to different PCR protocols and DNA per parasite estimations, sensitivities reported for the different *Leishmania* quantitative assays are generally not directly comparable. For example, Nicolas et al. (2002) reported detection of 0.1 parasite based on the assumption of 1 ng total DNA per *Leishmania* parasite, whereas it equates to less than 0.1 ng (∼84.3 fg) in the present study.

Mean *Leishmania* parasite numbers obtained by the absolute quantification method were consistent with parasite counts obtained by standard microscopic counting methods for infected sand flies from the same cohort. Parasite counts ∼8-fold higher than those observed by light microscopy were obtained using the Pfaffl (2001) relative quantification method based on the *Lu. longipalpis per* gene. However, it should be acknowledged that: (1) comparison between quantitative PCR and microscopy estimates for the same individual fly would have been more accurate; (2) further real-time replicate infected sand fly samples would increase the reliability of the assay; and (3) the efficiencies of *Leishmania DNA polymerase* and *Lu*. *longipalpis per* primers were not strictly comparable, precluding the use of the relatively simpler comparative, 2^−ΔΔCt^ method (Livak and Schmittgen, 2001). This indicates that other *Leishmania*/sand fly gene pairs should be explored, with emphasis on polymorphic single copy non-repetitive genes that could be also used for species identification. Further refinement of relative *Leishmania* quantification is worthwhile, because in contrast to the absolute quantification method, comparative methods are independent from the amount of sand fly DNA, optical density DNA concentration inaccuracies and the presence of PCR inhibitors.

Quantification of *Leishmania* live parasites in vertebrate host tissues using an18S rRNA-based quantitative PCR assay (QT-NASBA) with a chemiluminescent internal probe has been reported (Van der Meide et al., 2005). However, *Leishmania* DNA-based quantitative PCR appears to detect live parasites according to a recent multi-target real-time PCR study (Prina et al., 2007). Although the *Leishmania* DNA assay used in this study did not distinguish between *Leishmania* species or among differentiation stages within sand flies, intra-fly parasite densities obtained were consistent with those determined by other methods (Rogers et al., 2004, Warburg and Schlein, 1986). In the future, the development of a *Leishmania* stage-specific PCR assay would separately quantify the number of mammal-infective metacyclic and other non-infective stages within sand flies and in sand fly infective egested inoculum at the biting site. This is valuable information on the proportion of infective sand flies at any given time and on the influence of the infective inoculum size and composition on the vertebrate host's immune response. As effective parasite dose egested by sand flies at the bite site is likely to be determined by the co-evolutionary adaptive biochemistry of the association between *Leishmania* and sand fly genotypes, the method here described represents a first step towards the accurate estimation of metacyclogenesis rates when comparing relative vectorial efficiencies between different sibling sand fly species and in studies on *Leishmania* transmission mechanisms (Maingon et al., 2007, Montoya-Lerma et al., 2003, Rogers et al., 2004).

In conclusion, a highly reproducible quantitative PCR assay is described for estimating *Leishmania* numbers in sand flies, with intra-assay and inter-assay coefficient variations lower than 1 and 1.7%, respectively, and sensitivity down to 10 pg *Leishmania* DNA (∼120 parasites) in as much as 100 ng of sand fly DNA. Furthermore, estimated parasite loads within experimentally infected sand flies were within the range of counts obtained by microscopy for the same sand fly cohort or ∼five times higher than microscopy counts, depending on the method used for data analysis. These results highlight the potential of quantitative PCR and the need to further refine this powerful method for the study of natural sand fly infection rates and *Leishmania* transmission mechanisms.

## Funding

MER and PAB were supported by the Wellcome Trust (project grants 064945 and 078937).

## Conflicts of interest

None declared.

## Ethical approval

Not required.

## Authors’ note

Results herein reported are part of the thesis on ‘Chemical and molecular studies on *Leishmania* manipulation of *Lutzomyia longipalpis* blood feeding behaviour’ submitted by SR for the award of MPhil degree by Keele University, Staffordshire, UK. SR's research was co-supervised by JGCH and RDCM.

## Authors’ contributions

RDCM contributed to the experimental design and experimental work; SR produced all of the experimental work; MER and PAB contributed to the laboratory sand fly infection design and parasite counts; JGCH, MER and PAB provided experimental guidance; RDCM and SR analysed and interpreted the data; RDCM drafted the manuscript. All authors contributed to and read and approved the final manuscript. RDCM, SR and JGCH are guarantors of the paper.

## Appendix A Supplementary data

Supplementary Figure 1 Sensitivity of (A) *Leishmania* kDNA and (B) *DNA polymerase α* gene primers. Ethidium-bromide-stained PCR products separated on 1.75% (w/v) wide range agarose gels in Tris-acetate buffer for serial 10-fold dilutions of *Le. infantum* DNA (lanes 2–10) using (A) kDNA or (B) DNA polymerase primers. Lane 1: 50 bp DNA ladder (MBI Fermentas); lane 2: 100 ng; lane 3: 10 ng; lane 4: 1 ng; lane 5: 100 pg; lane 6: 10 pg; lane 7: 1 pg; lane 8: 100 fg; lane 9: 10 fg; lane 10: 1 fg; lane 11: molecular biology grade (MBG) water instead of *Leishmania* DNA. Sizes of marker fragments in base pairs are indicated on the left. The size estimated for the major PCR band is shown by an arrow on the right.

Supplementary Figure 2 Specificity of (A) *Leishmania DNA polymerase α* and (B) *Lutzomyia longipalpis periodicity* gene primers. Ethidium-bromide-stained PCR products separated on 1.75% (w/v) wide range agarose gels in Tris-acetate buffer alongside a 50 bp DNA ladder (Fermentas); lane 1 in both panels. (A) PCR with *Leishmania DNA polymerase* primers. Lanes 2–11: serial 10-fold dilutions of *Le*. *infantum* DNA (100 ng to 0.1 fg, respectively; each serial dilution contained 100 ng sand fly DNA); lane 12: molecular biology grade (MBG) water instead of *Leishmania* DNA; lane 13: 100 ng sand fly DNA. (B) PCR with *Lu*. *longipalpis per* primers. Lane 2: 100 ng sand fly DNA; lane 4: 100 ng *Leishmania* DNA; lane 5: 100 ng each *Leishmania* and sand fly DNA; lane 6: MBG water no template control; lane 3: 100 ng sand fly DNA with MS 6-001 primers. Sizes of marker fragments in base pairs are indicated on the left. The size estimated for the major PCR band is shown by an arrow on the right.
