# Supplementary material for: A real-time PCR assay to estimate Leishmania chagasi load in its natural sand fly vector Lutzomyia longipalpis
Source: Trans R Soc Trop Med Hyg. 2008 Sep;102(9):875–82. doi: 10.1016/j.trstmh.2008.04.003 (PMC2678673; doi:10.1016/j.trstmh.2008.04.003)
Supplement: Supplementary Figure 1 — Sensitivity of (A) Leishmania kDNA and (B) DNA polymerase α gene primers. Ethidium-bromide-stained PCR products separated on 1.75% (w/v) wide range agarose gels in Tris-acetate buffer for serial 10-fold dilutions of Le. infantum DNA (lanes 2–10) using (A) kDNA or (B) DNA polymerase primers. Lane 1: 50 bp DNA ladder (MBI Fermentas); lane 2: 100 ng; lane 3: 10 ng; lane 4: 1 ng; lane 5: 100 pg; lane 6: 10 pg; lane 7: 1 pg; lane 8: 100 fg; lane 9: 10 fg; lane 10: 1 fg; lane 11: molecular biology grade (MBG) water instead of Leishmania DNA. Sizes of marker fragments in base pairs are indicated on the left. The size estimated for the major PCR band is shown by an arrow on the right. [file mmc1.doc]

**Supplementary Table 1** *Leishmania* counts in 13 d post-infected female flies by microscopy: batch 1

| Fly number | Total parasite count | % metacyclics |
| --- | --- | --- |
| 1 | 97 897 | 93.5 |
| 2 | 98 498 | 56.4 |
| 3 | 75 075 | 17.6 |
| 4 | 21 621 | 60.9 |
| 5 | 144 744 | 71.8 |
| 6 | 14 414 | 2.8 |
| 7 | 15 615 | 93.9 |
| 8 | 330 330 | 72.9 |
| 9 | 22 822 | 66 |
| 10 | 40 240 | 43 |
| 11 | 600 | 9.1 |
| 12 | 43 243 | 9.7 |
| Mean count | 75 425 |  |
