# Supplementary material for: A real-time PCR assay to estimate Leishmania chagasi load in its natural sand fly vector Lutzomyia longipalpis
Source: Trans R Soc Trop Med Hyg. 2008 Sep;102(9):875–82. doi: 10.1016/j.trstmh.2008.04.003 (PMC2678673; doi:10.1016/j.trstmh.2008.04.003)
Supplement: Supplementary Figure 2 — Specificity of (A) Leishmania DNA polymerase α and (B) Lutzomyia longipalpis periodicity gene primers. Ethidium-bromide-stained PCR products separated on 1.75% (w/v) wide range agarose gels in Tris-acetate buffer alongside a 50 bp DNA ladder (Fermentas); lane 1 in both panels. (A) PCR with Leishmania DNA polymerase primers. Lanes 2–11: serial 10-fold dilutions of Le. infantum DNA (100 ng to 0.1 fg, respectively; each serial dilution contained 100 ng sand fly DNA); lane 12: molecular biology grade (MBG) water instead of Leishmania DNA; lane 13: 100 ng sand fly DNA. (B) PCR with Lu. longipalpis per primers. Lane 2: 100 ng sand fly DNA; lane 4: 100 ng Leishmania DNA; lane 5: 100 ng each Leishmania and sand fly DNA; lane 6: MBG water no template control; lane 3: 100 ng sand fly DNA with MS 6-001 primers. Sizes of marker fragments in base pairs are indicated on the left. The size estimated for the major PCR band is shown by an arrow on the right. [file mmc2.doc]

1 2 3 4 5 6 7 8 9 10 11

50

100

150

120

Origin

**A**

**B**

90

100

100

Origin

50

1 2 3 4 5 6 7 8 9 10 11
