# Supplementary figures and images for: A real-time PCR assay to estimate Leishmania chagasi load in its natural sand fly vector Lutzomyia longipalpis
Source: Trans R Soc Trop Med Hyg. 2008 Sep;102(9):875–82. doi: 10.1016/j.trstmh.2008.04.003 (PMC2678673; doi:10.1016/j.trstmh.2008.04.003)

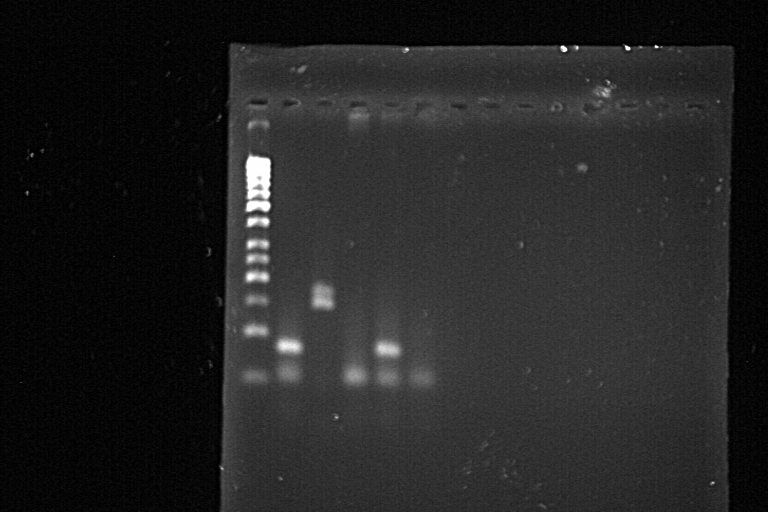


80

Origin

150

100

50

1 2 3 4 5 6

**B**

1 2 3 4 5 6 7 8 9 10 11 12 13

90

Origin

50

100

**A**

Supplement: Supplementary file 3 [file mmc3.doc]
